# Correlating the Effect of Composition and Textural Properties on Bioactivity for Pristine and Copper-Doped Binary Mesoporous Bioactive Glass Nanoparticles

**DOI:** 10.3390/ma16206690

**Published:** 2023-10-14

**Authors:** Florestan Vergnaud, Benhur Mekonnen, Abdelouahad El Abbassi, Charlotte Vichery, Jean-Marie Nedelec

**Affiliations:** Université Clermont Auvergne, Clermont Auvergne INP, CNRS, ICCF, F-63000 Clermont-Ferrand, France

**Keywords:** mesoporous bioactive glass nanoparticles, sol–gel, copper, apatite formation, composition, specific surface area

## Abstract

Multifunctional substitutes for bone tissue engineering have gained significant interest in recent years in the aim to address the clinical challenge of treating large bone defects resulting from surgical procedures. Sol–gel mesoporous bioactive glass nanoparticles (MBGNs) have emerged as a promising solution due to their high reactivity and versatility. The effect of calcium content on MBGNs textural properties is well known. However, the relationship between their composition, textural properties, and reactivity has not yet been thoroughly discussed in existing studies, leading to divergent conclusions. In this study, pristine and copper-doped binary MGBNs were synthesized by a modified Stöber method, using a cationic surfactant as pore-templating agent. An opposite evolution between calcium content (12–26 wt%) and specific surface area (909–208 m^2^/g) was evidenced, while copper introduction (8.8 wt%) did not strongly affect the textural properties. In vitro bioactivity assessments conducted in simulated body fluid (SBF) revealed that the kinetics of hydroxyapatite (HAp) crystallization are mainly influenced by the specific surface area, while the composition primarily controls the quantity of calcium phosphate produced. The MBGNs exhibited a good bioactivity within 3 h, while Cu-MBGNs showed HAp crystallization after 48 h, along with a controlled copper release (up to 84 ppm at a concentration of 1 mg/mL). This comprehensive understanding of the interplay between composition, textural properties, and bioactivity, offers insights for the design of tailored MBGNs for bone tissue regeneration with additional biological and antibacterial effects.

## 1. Introduction

Over the last years, there has been an increasing interest in multifunctional substitutes for bone tissue engineering. Despite having an intrinsic potential of self-healing in response to injury, the efficient treatment of large bone defects resulting from trauma, osteoporosis, or tumor resection still remains a clinical challenge. Moreover, bacterial infections can occur after surgical operations and hinder bone regeneration. Due to their deep localization, bone infections are difficult to treat. In the last decades, promising multifunctional materials have been proposed as bone regeneration systems. Indeed, the ideal bone substitute material should combine several abilities, including bioactivity, osteoconductivity, and stimulation of both osteogenesis and angiogenesis. In addition, an antibacterial effect could be provided by a local release of antibiotics or therapeutic ions.

To this aim, mesoporous bioactive glass nanoparticles (MBGNs) have attracted significant attention due to their ability to simultaneously enhance bone regeneration and provide biological effects. The formation of a hydroxyapatite (HAp) layer on their surface when implanted allows for a strong interfacial bond with the surrounding tissues. The ability to induce HAp crystallization (defined as bioactivity in the following) is mainly related to the glass calcium content and the MBGNs high specific surface area owing to their porosity. In addition, this unique textural property gives them the ability to efficiently release therapeutic agents such as antibiotics loaded in the pores or ions inserted in the glass matrix.

In recent years, copper-doped bioactive materials (including bioactive glasses, calcium phosphate ceramics, etc.) have emerged as promising candidates for bone tissue engineering owing to their ability to enhance bone regeneration and simultaneously induce angiogenesis and antibacterial effects thanks to copper release. Indeed, Jacobs et al. showed that copper-doped biphasic calcium phosphate powders had antibacterial effects even for Cu^2+^ concentrations of a few ppm and that the necessary concentration for bactericidity was strain-dependent (*Staphylococcus aureus*, *Escherichia coli*, and *Pseudomonas aeruginosa* were tested) [1]. This study also showed that a progressive copper ions release of 12 ppm is not cytotoxic. With a bioactive glass scaffold, Wu et al. measured up to 154 ppm of Cu in the cell culture medium after 7 days of immersion with no in vitro cytotoxic effect and with a significant antibacterial effect on *E. coli* [2]. Beneficial biological effects have also been demonstrated in the presence of this material, notably angiogenesis (better expression of the vascular endothelial growth factor VEGF) and osteostimulation (higher expression of osteopontin (OPN), osteocalcin (OCN), and alkaline phosphatase (ALP)). Copper-containing bioactive glass nanoparticles were also studied by Zheng et al. and showed a sustained release of copper ions with no cytotoxicity [3]. Several studies on copper-doped mesoporous bioactive glass nanoparticles (Cu-MBGNs) with a common nominal Cu content of 5%mol have shown promising results. Westhauser et al. reached a maximum concentration of released Cu^2+^ (18 ppm) after 48 h, and the presence of copper resulted in a significant increase in ALP expression with no negative effect on cell viability [4]. Bari et al. reported an antibacterial effect on different strains (*E. coli*, *S. aureus,* and *S. epidermidis*) with about 20 ppm of Cu^2+^ released in 7 days [5]. Finally, Hosseini et al. showed that a Cu^2+^ concentration between 30 and 35 ppm allowed for the inhibition of methicillin-resistant *S. aureus* growth after 24 h of exposure with, however, a cytotoxic effect at high powder concentrations [6]. A dose-dependent toxicity can thus be observed, which depends in particular on the method of evaluation [1,2]. With regard to the literature, the in vivo therapeutic window presenting a compromise between antibacterial, angiogenic, osteogenic, and cytotoxic effects, yet has to be identified [4].

Sol–gel synthesis is a promising approach to design multifunctional MBGNs. This soft-chemistry route, based on the hydrolysis and condensation of alkoxide precursors such as tetraethyl orthosilicate (TEOS) in basic aqueous/alcoholic solution at room temperature, provides a precise control on the size and morphology of bioactive glass (BG) particles and allows for the use of surfactants as pore-templating agents to obtain controlled porosity. Ca^2+^ and other elements of biological interest such as Cu^2+^ can be easily included in the silica glass, usually through the addition of salt precursors (nitrates and chlorides) during the synthesis.

The BGs bioactivity is usually studied through the evaluation of HAp crystallization kinetics and amount after immersion in simulated body fluid (SBF). It is a common and efficient preliminary test to assess for BG in vivo bioactivity [7]. The glass dissolution also leads to the release of doping ions, so there is a tight link between bioactivity and the doping ion concentration in the medium. It is important to mention that the BG composition and textural properties are strongly related, with Ca being known to affect NPs morphology and agglomeration and surfactant organization [8,9]. Several studies have already reported this interdependence (an increase in Ca content being detrimental to the specific surface area) and studied the HAp crystallization of samples with various compositions and specific surface areas in SBF [10,11,12,13]. However, few works extended the study to the coupled effect of these two parameters to unravel their respective roles on HAp formation.

Moreover, some studies’ conclusions are divergent regarding the predominant factor impacting bioactivity. De Oliveira et al. [10] compared binary bioactive glass micro- and nanoparticles with Ca/Si molar ratios of 0.68 and 0.22 and specific surface areas of 76.4 and 533.5 m^2^/g, respectively. Better-defined HAp Bragg peaks were observed for the NPs on XRD patterns, indicating that a high specific surface area was the factor with the most impact on apatite crystallization. Li et al. [11] observed a decrease in specific surface area proportional to the increase in calcium content (nominal Ca/Si ratio ranging from 0.01 to 0.92) for micrometric ternary glass particles. The composition was this time undoubtedly the predominant factor with respect to the crystallization rate and the amount of apatite formed. Martinez et al. [12] and Saravanapavan et al. [13] studied binary glasses (as micrometric particles and monoliths, respectively) whose nominal composition varied between 91S9C and 52S48C (S = wt% SiO_2_ and C = wt% CaO). In both cases, the increase in calcium content was associated with a sharp decrease in specific surface area (up to 5 to 6 times lower). Immersion tests in SBF showed a higher rate of HAp formation with increasing calcium content. In both studies, however, the 50S50C composition either mainly induced calcite crystallization [12] or slowed down apatite crystallization [13], indicating that calcium in excess may be detrimental to bioactivity. All the former studies highlighted the complex relationship between bioactivity and the bioactive glass composition and specific surface area and sometimes the need for a compromise. Furthermore, the lack of systematic evaluation of the glasses’ actual composition by elemental analysis in most of these studies complicates the comparison. The various synthesis routes employed indeed usually lead to a discrepancy between glasses’ nominal (linked to the precursor amounts) and effective compositions. In order to uncouple the effect of composition and textural properties on bioactivity, our team’s previous work focused on obtaining dense bioactive glass nanoparticles of variable composition (Ca/Si effective molar ratio of 0.18, 0.12, and 0.07) with a similar specific surface area (19 ± 1 m^2^/g) [14]. As the impact on HAp amount and crystallization kinetics was low in the compositional range studied, it was assumed that the bioactivity should be rather improved by achieving higher specific surface areas. We thus aim to complement the aforementioned work by performing a similar study on MBGNs. In this paper, a “better” bioactivity refers to faster HAp crystallization kinetics, and a larger amount of formed HAp crystals, keeping in mind that the ideal situation for bone regeneration is, however, to match the BG degradation rate to that of biological processes [15].

Regarding Cu-MBGNs, the substitution of calcium by such a doping ion, which is not necessary for bioactivity mechanisms and HAp formation, has led to contradictory results [16,17]. Zheng et al. observed HAp crystals on copper-doped BG after 3 days in SBF, whose amount is related to Cu content, while no HAp was observed on undoped BG [3]. On the contrary, Bejarano et al. reported a decrease in reactivity for Cu-doped 58S glass, and Hosseini reported after 1 day in SBF the absence of HAp with Cu-MBGNs, while crystallization was evidenced for undoped MBGNs [6,18]. Some studies reported no copper influence, as Bari et al. evidenced HAp precipitation after only 3 h of immersion for both doped and undoped MBGNs, and Baino et al. reported similar results after 14 days in SBF for various copper amounts [5,19]. The effect of inserted Cu^2+^ in the bioactive glass matrix on HAp crystallization thus remains controversial.

Therefore, the objective of this work is to unravel the individual or coupled impact of composition and textural properties of MBGNs on their bioactivity. To do so, undoped and Cu-doped binary MGBNs were synthesized by a modified Stöber method, using a surfactant as a pore-templating agent. Calcium content was gradually increased, copper was added, and their effects on structural and textural properties were reported through an in-depth characterization of the materials. Subsequent bioactivity assays of MBGNs and Cu-MBGNs were carried out in SBF to allow for a thorough analysis of glass reactivity, i.e., HAp formation rate and amount, and copper release.

## 2. Materials and Methods

### 2.1. Materials

Tetraethyl orthosilicate (TEOS, 99%), cetyltrimethylammonium bromide (CTAB, 98%), ammonium hydroxide solution (NH_3_, 28–30%), calcium nitrate tetrahydrate (Ca(NO_3_)_2_·4H_2_O, 99%), and copper nitrate trihydrate (Cu(NO_3_)_2_·3H_2_O, 99–104%) were purchased from Sigma-Aldrich (St. Louis, MO, USA). Absolute ethanol (EtOH, 99.5%) was obtained from VWR Chemicals. Deionized water was used in all experiments.

### 2.2. Synthesis of MBGNs and Cu-MBGNs

Mesoporous bioactive glass nanoparticles (MBGNs) and copper-doped mesoporous bioactive glass nanoparticles (Cu-MBGNs) were synthesized through a modified Stöber method, using CTAB as pore-forming agent. Briefly, 430 mg of CTAB was dissolved in a solution containing 209.5 mL of H_2_O and 87.6 mL of EtOH and left under vigorous stirring at room temperature until complete dissolution. Afterward, 3.13 mL of concentrated ammonia was poured into the solution, followed by the addition of 1.4 mL of TEOS. The resulting mixture was maintained under stirring for 3 h. Then, a given amount of Ca(NO_3_)_2_·4H_2_O dissolved in 2.5 mL of H_2_O was added, and the resulting solution was maintained under stirring for 20 h. The white precipitate was collected by centrifugation (6297× *g* for 10 min) and washed three times with deionized water and once with ethanol to remove unreacted chemicals. The particles were dried overnight at 60 °C, and a final annealing step (5 °C/min for 3 h at 350 °C followed by 3 h at 650 °C) allowed for CTAB decomposition and Ca^2+^ ions’ incorporation into the silica network. Four MBGNs samples named MBGN-X were obtained, corresponding to initial Ca/Si molar ratios of X = 0.25, 0.5, 1, and 2 (0.37, 0.74, 1.49, and 2.98 g of calcium nitrate, respectively). For the Cu-MBGN sample, the synthesis protocol was identical to that of MBGN-1, with one additional step: 0.15 g of Cu(NO_3_)_2_·3H_2_O dissolved in 2.5 mL of water was added 30 min after calcium addition (initial Cu/Si molar ratio of 0.1).

### 2.3. Morphological and Structural Characterizations

Nanoparticles morphology and size distribution were observed by transmission electron microscopy (TEM, Hitachi H-7650 operating at 80 kV) on powder samples previously dispersed in deionized water by sonication. First, 10 µL of the suspensions were deposited on carbon–formvar copper grids and left for drying overnight at room temperature. At least 200 particles were measured using the ImageJ software (v 1.54d) to determine particle size distribution. X-ray diffraction (XRD) patterns were recorded in the 2θ range 20–50° with a step of 0.016 ° using a D2 phaser (Bruker, Billerica, MA, USA) diffractometer working in Bragg–Brentano configuration with a Cu anode (λ_Kα1_ = 1.5406 Å, λ_Kα2_ = 1.5444 Å). Infrared spectra were acquired by Fourier-transform infrared spectroscopy (FTIR) using Thermo Scientific Nicolet 5700, iS10 or Summit Pro spectrometers (Thermo Fisher Scientific. Waltham, MA, USA) in transmission mode on KBr pellets. The weight ratio KBr:sample was about 199:1. The sample composition was determined by energy-dispersive X-ray spectroscopy (EDS) using a Hirox SH-4000M scanning electron microscope (Hirox Europe, Limonest, France) with Bruker XFlash Detector 630M (Bruker, Billerica, MA, USA) or by inductively coupled plasma–atomic emission spectroscopy (ICP-AES) using an Agilent 5800 spectrometer (Agilent, Santa Clara, CA, USA). For EDS measurements, samples were pressed into pellets and deposited on a carbon film. For ICP-AES measurements, sample preparation and reference materials are described in detail in a previous article [20]. The analytical wavelengths used for Cu, Si, and Ca are λ = 218.598 nm, λ = 251.611 nm, and λ = 317.933 nm, respectively. Nitrogen adsorption–desorption isotherms were recorded with a Micromeritics Tristar II PLUS sorptometer. The samples’ specific surface area and pore size distributions were determined by the Brunauer–Emmett–Teller (BET, calculated in the 0.05–0.3 P/P^0^ range) and Barrett–Joyner–Halenda (BJH) methods, respectively. Pore volume was calculated at P/P^0^ = 0.95. ^29^Si magic angle spinning–nuclear magnetic resonance (MAS-NMR) spectra were acquired on a Bruker Advance300 spectrometer under a magnetic field of 7.05 T using a 4 mm probe. Experiments were performed on fine powders filled in 4 mm zirconia rotors spinning at 10 kHz. The recorded spectra were obtained summing approximately 2400 scans. The chemical shifts were referenced to TEOS (signal located at −86.04 ppm of TMS, tetramethylsilane). A pulse length of 5.10 μs was applied with a relaxation delay of 60 s.

### 2.4. In Vitro Bioactivity Study

The samples’ ability to form hydroxyapatite when in contact with body fluids was assessed in vitro. Prior to the assays, simulated body fluid (SBF), an aqueous saline solution that mimics the pH and ionic concentrations of human blood plasma, was prepared according to the method described by Kokubo et al. [7]. The MBGNs or Cu-MBGNs powders were soaked in SBF (1 mg.mL^−1^) in a plastic beaker and kept in an orbital shaking incubator (N-BIOTEK, NB-205) at 37 °C for 3, 24, 48, or 96 h. For each immersion time, the whole sample was recovered by centrifugation, gently washed twice with deionized water, and dried at 60 °C. The formation of hydroxyapatite crystals was subsequently evidenced by XRD, FTIR, and electron microscopy. To specifically study hydroxyapatite crystals derived from Cu-MBGNs particles, a Hitachi Regulus 8230 field-emission SEM (30 kV) equipped with a STEM detector (scanning transmission electron microscopy) for imaging, and an Oxford Ultim Max 170 mm^2^ detector for microanalysis was used. The sample was prepared on a nickel grid to avoid copper interference, following the same method as for other TEM analyses. Three EDS spectra were obtained by focusing either on apatite needles or on spherical bioactive glass NPs.

### 2.5. Copper Release in SBF

In addition to hydroxyapatite mineralization, copper release from Cu-MBGNs was assessed thanks to the same experiment in SBF. After a given immersion time, alongside powder recovery, the supernatant was collected and filtered through a 0.2 µm filter. The copper concentration in the latter was quantified by ICP-AES using an Agilent 5800 spectrometer using an analytical wavelength of λ = 327.395 nm for Cu.

## 3. Results and Discussion

### 3.1. MBGNs Morphological and Structural Characterizations

#### 3.1.1. Particle Size and Morphology

**Figure 1:**
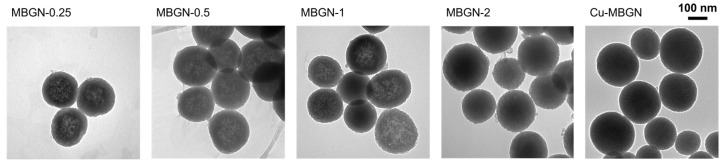
TEM images of MBGNs with different amounts of calcium (Ca/Si ratios from 0.25 to 2) and of Cu-MBGNs.

The morphology of MBGNs synthesized with different amounts of calcium (initial Ca/Si ratio ranging from 0.25 to 2) and Cu-MBGNs (initial ratios Ca/Si = 1 and Cu/Si = 0.1) was observed by transmission electron microscopy. Figure 1 shows that all the MBGNs present a spherical and non-agglomerated morphology regardless of the amount of Ca or Cu precursors. MBGNs size distribution remained relatively unchanged, with mean sizes of 198 ± 17, 211 ± 20, 188 ± 21, 199 ± 25 nm, and 148 ± 29 nm, obtained by a log-normal fitting of size histograms (Figure S1 in Supplementary Materials) for MBGN-0.25, 0.5, 1, 2, and Cu-MBGN samples, respectively. No evident pore organization is visible on the micrographs, suggesting a rather wormlike pore structure due to Ca^2+^ addition, as already shown in previous studies [9,21].

Apart from a slight increase in size dispersion (from 9 to 13%), no trend in average size was evidenced for the MGBNs samples in contrast to what has sometimes been reported in the literature [21]. The smaller particle size for Cu-MBGNs could be explained by the change in EtOH/H_2_O ratio during the synthesis, which is caused by the addition of water for copper introduction.

#### 3.1.2. Structural Studies

**Figure 2:**
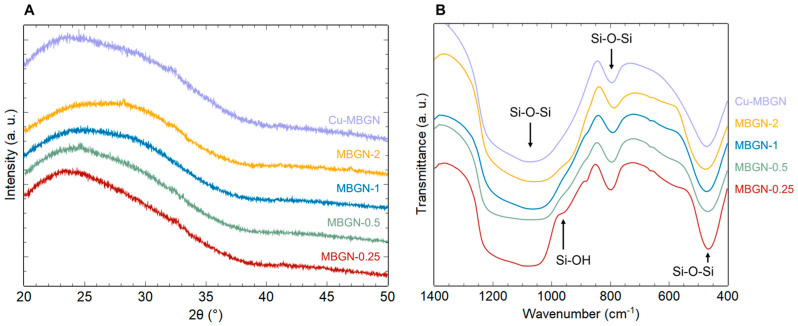
XRD patterns (**A**) and FTIR spectra (**B**) of MBGNs with Ca/Si ratios ranging from 0.25 to 2 and Cu-MBGNs.

X-ray diffractograms on Figure 2A show the typical amorphous halo corresponding to silica glass. As no Bragg peaks stand out from the background, no secondary phases such as calcium silicate, calcium carbonate, or copper oxide (for Cu-MBGNs) are evidenced, meaning they are either inexistent, poorly crystallized, or in very small amounts. Figure 2B shows the superposition of the IR spectra of the different samples. Four adsorption bands can be observed between 400 and 1300 cm^−1^, characteristic of the silica matrix [22]. The bands located at 450–490 cm^−1^ and at 760–850 cm^−1^ can be associated with deformation vibrations of Si-O-Si bonds. The band located between 1000 and 1300 cm^−1^ corresponds to the asymmetric elongation vibration of Si-O-Si bonds and the one around 900–980 cm^−1^ to the symmetrical elongation vibration of Si-OH (residual silanol groups inherent to the sol–gel route). The weak band around 860–880 cm^−1^ may correspond to O-C-O bonds (possible unwanted calcium carbonates).

In summary, infrared spectroscopy and X-ray diffraction results indicate that neither the increasing initial Ca/Si ratio nor the presence of copper induced significant structural changes. Increasing amounts of calcium and copper were incorporated into the silica matrix as network modifiers without secondary phase crystallization. However, this only applies to a limited range of copper doping: Copper oxide (CuO) and calcium silicates were evidenced for an initial ratio Cu/Si > 0.1, probably due to Coulombic repulsion between Cu^2+^ and Ca^2+^ ions, as previously reported by Bejarano et al. [18]. Although the introduction of a lower copper content is feasible (Figure S2 in Supplementary Materials), this study focuses on investigating copper release from the Cu-MBGN sample with the maximum copper doping without CuO crystallization.

#### 3.1.3. MBGNs and Cu-MBGNs Composition

Calcium incorporation was confirmed by EDS. However, this technique did not enable accurate copper quantification. Effective Cu-MBGN composition was therefore measured by ICP-AES. The results of the elemental analysis are highlighted in Table 1. The evolution of measured calcium proportion for MBGNs is consistent with the initial precursor quantities, but a linear proportional relationship cannot be established between the nominal and the effective amount of calcium in the samples. ICP-AES measurement of Cu-MBGN confirms that most of the copper was incorporated in the silica matrix (effective molar ratio Cu/Si = 0.09 ± 0.01) at the expense of calcium incorporation. Indeed, the measured Ca/Si ratio for Cu-MBGN is slightly lower than the one of MBGN-1, while both were synthesized with an initial ratio Ca/Si = 1. Hence, for a given amount of silica, the incorporable cation quantity seems to be limited, probably due to the finite number of adsorption sites on particles’ surface and the washing steps, which removed some of the adsorbed Ca^2+^ and Cu^2+^. This saturation was already documented with calcium for dense bioactive glass NPs obtained by the Stöber route [8,23]. It is worth mentioning that the maximum amount of calcium incorporated in this study is about three times greater than the previously mentioned references thanks to the use of CTAB, which allowed a significant increase in adsorption sites, as shown hereafter by the textural properties.

#### 3.1.4. Particles Textural Properties

**Figure 3:**
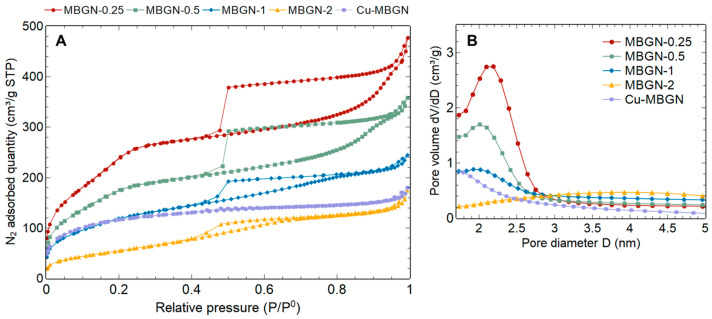
N_2_ adsorption isotherms (**A**) and pore size distributions (**B**).

Type IV adsorption isotherms, which are characteristic of mesoporous materials, are evidenced in Figure 3A. The abrupt desorption at a very specific relative pressure (P/P^0^ between 0.4 and 0.5) is typical of the nitrogen cavitation phenomenon. The pore size distributions were therefore deduced from the adsorption branches and are plotted for each sample in Figure 3B. Calculated specific surface area (S_BET_) and pore volume (V_BJH_) are summarized in Table 1, and a significant trend can be observed. The increase in calcium content clearly induces a decrease in pore volume and specific surface area. It should be noted that S_BET_ and V_BJH_ variations are inversely proportional to effective Ca/Si ratio variations (linear fits with R^2^ > 0.9). The pores being roughly of the same size for all MBGNs, this decrease could be explained by a lower pore number caused by the interactions between Ca^2+^ ions and the surfactant CTA^+^ polar heads. Comparing MBGN-1 and Cu-MBGN, the textural properties are slightly affected by Ca^2+^ substitution by Cu^2+^. Cu-MBGN pore size distribution seems to indicate a greater proportion of smaller-sized pores, which is coherent with the measured lower pore volume and similar specific surface area (higher pore surface/volume ratio).

This strong influence of calcium content suggests several possible explanations. First, Ca^2+^ ions appear to adsorb not only on the NPs surface during synthesis but also in the internal spaces of the silica particles, even when occupied by surfactant molecules. The theoretical specific surface area of a dense silica sphere with an average diameter of 200 nm is about 15 m^2^/g. This value is comparable to the S_BET_ reported for dense nanoparticles by Kesse et al. (19 m^2^/g), for which the maximum Ca/Si ratio measured was ~0.10 with excess calcium in the synthesis medium and after Ca(NO_3_)_2_ addition at a similar time of 3 h after TEOS [8]. This suggests that here, the adsorption sites that allowed much higher Ca/Si ratios are internal to the mesostructured particles. On the other hand, the strong variation of the textural properties as a function of the calcium ratio indicates that the structure is not yet rigid 3 h after the start of the sol–gel reaction, when Ca^2+^ is added to the medium and interacts with the surfactant “inside the pores”. Therefore, pore reorganization or collapse could occur just after Ca^2+^ addition, causing the variations discussed above. When the CTAB concentration is very low (1 mM), and the initial Ca/Si ratio is relatively high (0.5), the particles do indeed collapse and lose their spherical shape, which would be consistent with the above-mentioned hypothesis (Figure S3 in Supplementary Materials).

The specific surface area values obtained here are among the highest in the literature for the initial amounts of calcium used. Yun et al. were able to obtain one of the highest-documented values of 1040 m^2^/g for a ternary SiO_2_-CaO-P_2_O_5_ bioactive glass with a nominal Ca/Si molar ratio of 0.21 but with significant NPs agglomeration [21]. Numerous studies have reported the synthesis of MBGNs with an effective Ca/Si ratio of up to 0.2. Compared to our work, either a lower S_BET_ was obtained for a comparable calcium level, or conversely, a similar or higher specific surface area was achieved at the expense of Ca insertion in the silica matrix [24,25,26,27,28]. Although certain works did not measure the actual composition, it is nevertheless observed to follow a similar trend [21,29]. Analogous observations are made regarding the pore volume. Thus, our spherical, non-agglomerated MBGNs appear promising in the light of the reported specific surface area and pore volume results for similar systems, showing a good compromise between their textural properties and calcium content.

Regarding copper-containing particles (Cu-MBGNs), many studies reported a significant decrease in specific surface area, which was not observed here. Indeed, when 0 to 5 mol% CuO were substituted for CaO, Wu et al. [2] found a drop in specific surface area from 439 to 334 m^2^/g on a hierarchically porous material, while Baino et al. [19] reported a loss from 450 to 275 m^2^/g on a porous bioactive glass. For Cu-MBGNs, Bari et al. [5] observed a decrease from 621 to 224 m^2^/g; however, Hosseini et al. [6] showed a slight increase from 49 to 77 m^2^/g with the addition of copper salt. The former added Ca and Cu salts before TEOS, whereas the latter employed a similar synthesis to the present study (adsorption of Ca^2+^ and Cu^2+^ after NPs formation). We can infer that the organization of the mesophase during our synthesis was already strongly affected by the presence of calcium salt (see Table 1), and the impact of a subsequent addition of copper nitrate was therefore minimal.

#### 3.1.5. Network Connectivity

**Figure 4:**
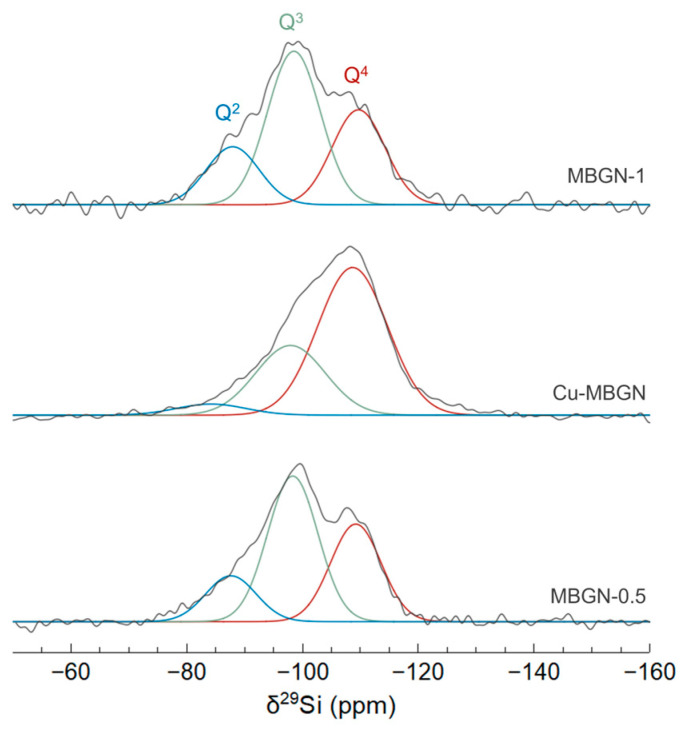
Experimental ^29^Si MAS-NMR spectra of MBGN-1, MBGN-0.5, and Cu-MBGN samples (grey lines, arbitrary units) fitted with Gaussian functions corresponding to Q^4^ (red), Q^3^ (green), and Q^2^ (blue) environments.

In order to gain a better understanding of the reactivity of the different samples when immersed in SBF, their network connectivity was evaluated by studying the local environment of silicon through solid-state ^29^Si MAS-NMR spectroscopy. Solubility is indeed strongly affected by the glass network connectivity, which is expected to be strongly correlated with the amount of calcium. MBGN-0.5, Cu-MBGN, and MBGN-1 spectra (ranked here in order of increasing measured calcium content) are shown in Figure 4.

Spectral deconvolution was necessary to identify the various Q^n^ environments present in the silica network, where Q represents a SiO_4_ tetrahedron and n (0 < n < 4) the number of bridging oxygens between two neighboring silicon atoms. The spectra were deconvoluted using a sum of three Gaussian functions. Three chemical shifts can be observed, which can be assigned to Q^4^, Q^3^, and Q^2^ species in the ranges [–110 to –108 ppm], [–99 to –98 ppm], and [–89 to –84 ppm], respectively [30]. The relative populations are given in Table 2. The evolution between MBGN-0.5 and MBGN-1 is consistent with their effective Ca/Si ratios. Indeed, Ca^2+^ ions incorporated into the glass network act as network modifiers and induce the creation of non-bridging oxygens (NBOs) and Si-O-NBO^−^ bonds at the expense of Si-O-Si bonds, with the NBOs negative charge being compensated by Ca^2+^ ions [31]. With an increase in the Ca/Si ratio from 0.22 to 0.30, the proportion of Q^4^ decreases in favor of Q^2^, which results in a slightly lower connectivity (quantified by the coordination number CN = 4 × %Q^4^ + 3 × %Q^3^ + 2 × %Q^2^ + 1 × %Q^1^).

Regarding the Cu-doped MBGN sample, which has an intermediate Ca/Si ratio of 0.25, the Si environment is clearly different, indicating a significantly higher Q^4^ content and therefore a higher CN, while we would have expected an intermediate CN. Copper incorporation thus seems to be directly responsible for this increase in connectivity, indicating that copper could act here as a network former, as has already been previously reported [32,33]. Other works observed a similar increase in connectivity with copper-doped bioactive glass and suggested a network repolymerization effect caused by the copper incorporation and CuO formation [34,35]. The more covalent nature of the Cu-O bond compared to the Ca-O bond could induce this repolymerization of NBOs. This hypothesis is consistent with the observation of CuO nanocrystals detected for higher copper content (see Figure S2 in Supplementary Materials). This increased connectivity could have an impact on the doped glass reactivity once immersed in SBF.

### 3.2. In Vitro Bioactivity in SBF

#### 3.2.1. Reactivity of Binary Bioactive Glass Nanoparticles (MBGNs)

As detailed previously through material characterization, the increase in the calcium content of MBGNs is inevitably accompanied by a degradation of textural properties. The four binary glasses, namely MBGN-0.25, 0.5, 1, and 2, were thus investigated after immersion in SBF (for 3 h and 24 h) to identify which factor, i.e., either %Ca or S_BET_, should be prioritized to maximize reactivity. These samples will be referred to in the following part by their mass composition measured by EDS, namely 88S12C, 83S17C, 78S22C, and 74S26C (where S = wt%SiO_2_ and C = wt%CaO), to reflect their actual composition more accurately than their initial Ca/Si ratio.

**Figure 5:**
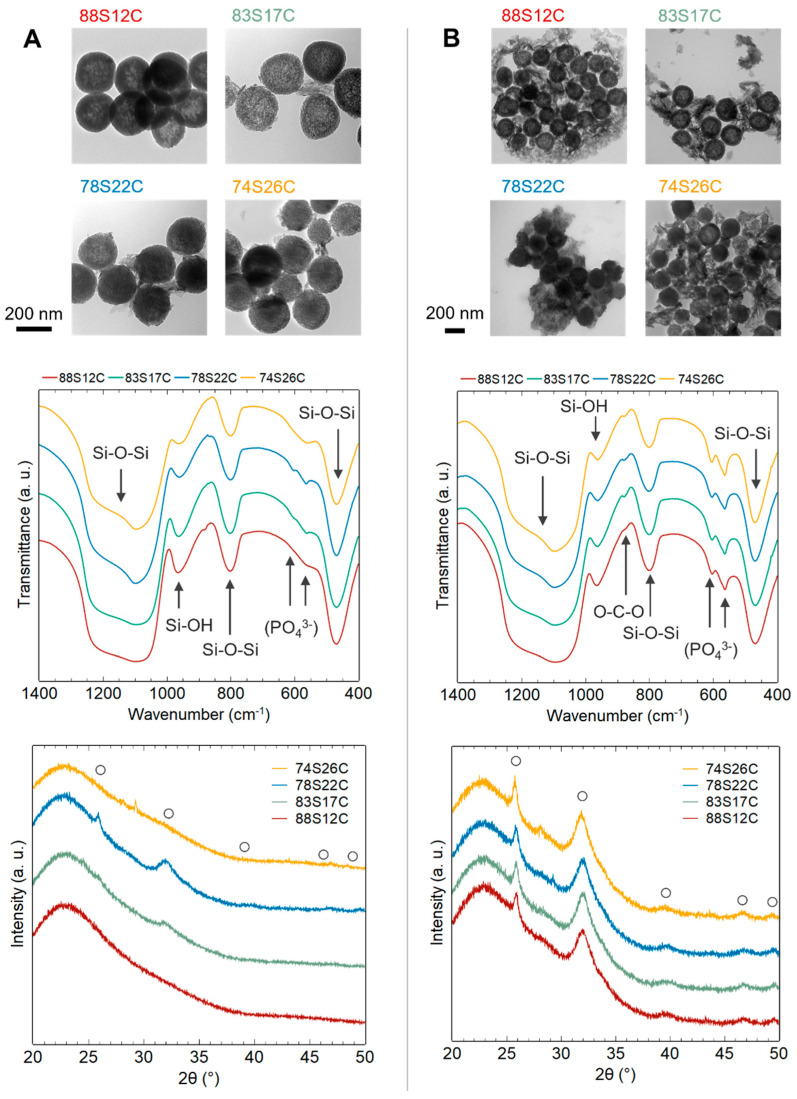
TEM images, FTIR spectra, and XRD patterns of MBGNs after 3 h (**A**) and after 24 h (**B**) of immersion in SBF (○, hydroxyapatite JCPDS 09-0432).

For a given immersion time of 3 h (Figure 5A), a greater amount of apatite seems to have crystallized as the calcium content was increased, according to the increasing HAp X-ray diffraction peak intensities. However, the sample with the highest calcium content (74S26C) differs from this trend, as no crystalline phase was detected by XRD. However, FTIR spectra provide additional information to this observation: After 3 h of immersion, all samples displayed absorption bands corresponding to the vibration of phosphate groups. Despite the absence of diffraction peaks, the bioactivity mechanism was therefore initiated for all samples.

An amorphous, phosphate, and carbonate-rich layer is indeed a prerequisite for the crystallization of carbonated hydroxyapatite, according to the bioactivity mechanism proposed by Hench [36]. For the outer compositions (88S12C and 74S26C), the formation of this layer after 3 h in SBF is characterized by the presence of a single wide band around 570 cm^−1^, corresponding to the antisymmetric vibration of the P-O bond of amorphous calcium phosphates [37,38]. For the intermediate compositions (83S17C and 78S22C), a doublet at 565 and 601 cm^−1^ characteristic of crystalline calcium phosphates appeared [37,38]. The band at 879 cm^−1^ corresponds to CO_3_^2−^. Its intensity could be correlated with the variation in specific surface area between samples (ranging from 909 m^2^/g for 88S12C to 208 m^2^/g for 74S26C) and thus the number of potential carbonate adsorption sites from the SBF. In summary, the 88S12C sample is only in an early stage of apatite formation after 3 h of immersion in SBF. For the same immersion time, increasing the calcium content resulted at the first sight in the crystallization of a greater amount of apatite. However, for the sample with the highest calcium content (74S26C), the mineralization process seemed to be inhibited, as indicated by the absence of HAp Bragg peaks. The reduction in the specific surface area correlated with the increasing %Ca therefore has a significant impact on the early stages of the bioactivity mechanism. However, after 24 h of immersion (Figure 5B), all samples exhibited a large amount of crystallized HAp, as shown by TEM images (needle-like particles) and XRD patterns, and very few differences are observable without further analysis.

To precisely understand the simultaneous effect of calcium content and specific surface area variations, the amount of calcium phosphate (CaP) was semi-quantitatively evaluated from the infrared spectra. To achieve this, the areas under the bands between 545 and 650 cm^–1^ corresponding to the vibration of phosphate groups (A_P–O_) and under the entirety of the spectrum between 400 and 840 cm^–1^ (A_TOT_) were determined. The A_P–O_/A_TOT_ ratio was then calculated for each sample at a given immersion time (0, 3, and 24 h), providing a semi-quantitative indication of the amount of hydroxyapatite (HAp) formed over time.

**Figure 6:**
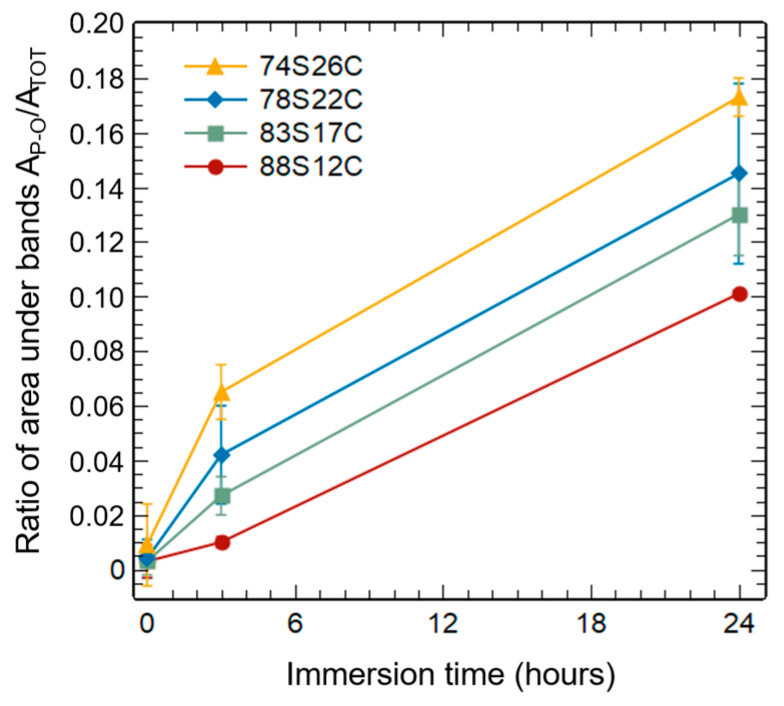
Ratio of the area under P-O-P bands (A_P-O_) over total area (A_TOT_) for different immersion times.

As shown in Figure 6, the amount of CaP formed at a given time appears to be strongly linked to the material composition. Indeed, regardless of the immersion time, a stronger proportion of calcium phosphates is observed for an increasing calcium content in the MBGNs. The 74S26C sample therefore allowed for the formation of the largest quantity of calcium phosphates (amorphous CaP, then crystalline HAp) despite a slowed crystallization into hydroxyapatite at the beginning of the mineralization process.

Based on these results, it appears that both composition and specific surface area govern distinct phenomena in the bioactivity mechanism. The calcium content, by reducing the connectivity of the glass network, allows for a rapid dissolution of the glass and the formation of a larger amount of calcium phosphate (amorphous or crystallized apatite). On the other hand, the exchange surface between the material and the medium influences the crystallization kinetic of this amorphous layer into hydroxyapatite based on the number of favorable sites for apatite nucleation. These results evidence why sample 78S22C (MBGN-1), which showed the best compromise between the crystallization rate and amount of HAp formed, served as the starting material to produce Cu-MBGNs.

#### 3.2.2. Reactivity of Copper-Doped Bioactive Glass Nanoparticles (Cu-MBGNs)

**Figure 7:**
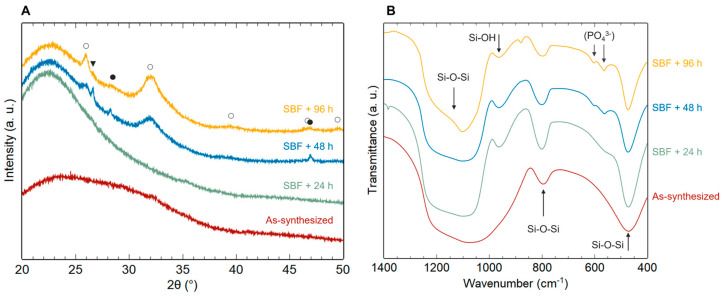
XRD patterns (**A**) and FTIR spectra (**B**) of as synthesized Cu-MBGNs and after 24, 48, and 96 h of immersion in SBF (○, hydroxyapatite JCPDS 09-0432; ●, calcium carbonate 47-1743; ▼, potassium magnesium silicate 82-0547).

Cu-MBGNs bioactivity was evaluated for different immersion times in SBF (Figure 7). Surprisingly, no diffraction peaks were observed after 24 h of immersion, whereas undoped samples exhibited intense peaks (Figure 5B). The absorption band corresponding to amorphous calcium phosphates was slightly visible after 24 h in SBF (Figure 7B), indicating that bioactivity mechanisms were initiated despite a delayed HAp crystallization.

The effect of copper introduction on bioactive glass reactivity is still quite controversial in the literature [17]. Here, Cu^2+^ has a visible impact on hydroxyapatite crystallization, as it is necessary to wait between 24 and 48 h to observe HAp peaks on XRD patterns. This bioactivity kinetics inhibition is not systematically observed after copper doping but has already been reported in some studies [6,39,40]. It could be explained by several factors. On one hand, the higher glass connectivity for Cu-MBGNs (evidenced in this study by ^29^Si MAS-NMR) could reduce the glass dissolution rate and therefore slow down the bioactivity process [34]. On the other hand, copper release into the medium could induce a competition between Ca^2+^ and Cu^2+^ ions for calcium phosphate precipitation and have a negative effect on apatite crystallization [18,40]. This hypothesis about the slight inhibition of bioactivity by copper is supported by the observation of intense diffraction peaks corresponding to hydroxyapatite as early as 24 h in SBF for samples with a lower copper content (initial Cu/Si molar ratio = 0.1–0.5; Figure S4 in Supplementary Materials). For these samples, apatite crystallization seems less affected due to a lower copper content.

#### 3.2.3. Copper Release in SBF

Similarly to Ca^2+^ ions, which diffused in the glass network during annealing, as demonstrated in many studies [8,31,41], copper ions should be homogeneously distributed in the particles. It is thus expected that Cu^2+^ ions will be gradually released upon the dissolution of MBGNs immersed in a biological fluid. We therefore quantified copper concentration in the solution after different immersion times of the Cu-MBGNs sample in SBF.

**Figure 8:**
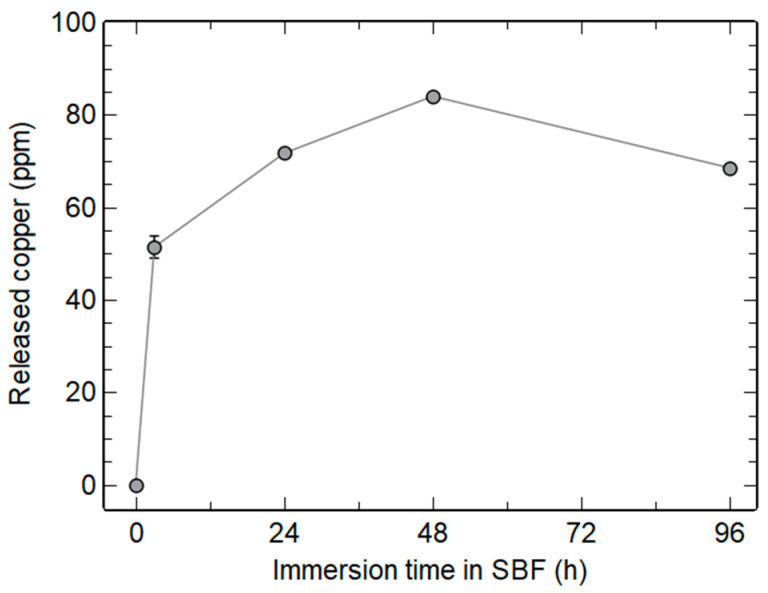
In vitro Cu^2+^ release profile as a function of immersion time in SBF (powder concentration 1 mg/mL).

Figure 8 shows a rapid release of copper in SBF, with 51 ppm detected within 3 h, increasing up to 84 ppm after 48 h. This amount appears to be within the desired therapeutic window. Indeed, copper concentrations ranging from 12 ppm to 154 ppm led to angiogenic, bacteriostatic, or bactericidal effects with no significant cytotoxicity [1,2,4,5]. However, Hosseini et al. showed a cytotoxic effect at high powder concentrations, probably due to the accumulation of the ions released under static conditions [6]. In the case of the present study, the amount of copper can be controlled by the glass doping to limit material toxicity. Indeed, Cu-MBGNs showed a Cu^2+^ release proportional to the initial Cu/Si ratio used for their synthesis (Figure S5 in Supplementary Materials).

Interestingly, a decrease in the amount of Cu in the medium between 2 and 4 days in SBF is evidenced in Figure 8. A similar trend was reported by Aina et al. between 7 and 14 days [40]. In addition, Hoppe et al. identified copper in the newly formed calcium phosphate layer by chemical mapping [34]. Incorporation of copper in the hydroxyapatite crystals during their formation could thus explain the decrease in [Cu^2+^] in solution. To verify this hypothesis, EDS spectroscopy was performed on both apatite needles and Cu-MBGNs using a STEM.

**Figure 9:**
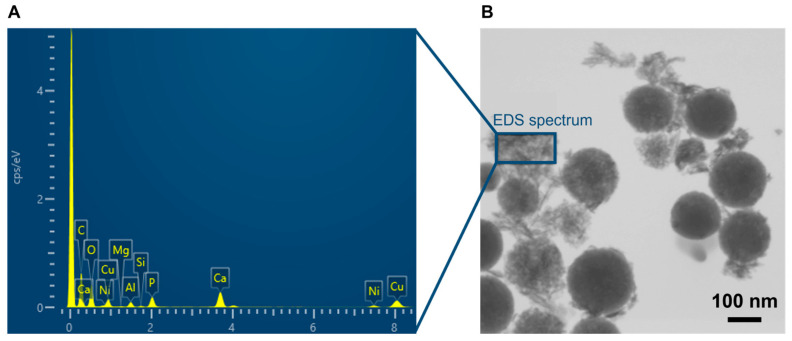
EDS spectra (**A**) of apatite crystals identified on a STEM image of Cu-MBGNs after 4 days in SBF (**B**).

Figure 9A shows the presence of phosphorus, with an average Ca/P ratio of 1.676 ± 0.169, which is coherent with the hydroxyapatite nature of the crystals (theoretical ratio of 1.667). Copper presence in or on the apatite crystals is also evidenced on the EDS spectrum. Spectra focusing on as-synthesized Cu-MBGNs particles and on the Cu-MBGNs particles after 4 days in SBF (Figure S6 in Supplementary Materials) reveal a decrease in Ca and Cu levels after immersion (loss of nearly 100% of Ca and 63% of Cu), consistent with Hench’s bioactivity mechanisms, where the bioactive glass is depleted in alkali and doping ions during mineralization [36]. On dense monolithic samples, these phenomena remain mostly superficial. However, the nanometric and porous nature of our particles seems to have allowed for an almost complete extraction of calcium from the glass. The residual amount of copper in the glass could be explained by the more covalent nature of the Cu-O bond compared to the Ca-O bond. Copper insertion in the HAp crystals unfortunately could not be confirmed by Rietveld analysis because of the nanometric and weakly crystallized aspect of the newly formed biological-like apatite, leading to an important XRD peak broadening. However, it is known that HAp can easily accommodate copper doping via an insertion mechanism [1,42,43]. Further investigations would be needed to prove whether copper ions are adsorbed onto the HAp crystals or inserted into their crystalline structure.

## 4. Conclusions

Highly bioactive MBGNs with different amounts of Ca^2+^ and Cu^2+^ were synthesized to assess the coupled effect of composition and textural properties on in vitro reactivity. For all binary MBGNs, the high specific surface areas (208–909 m^2^/g) coupled with a sufficient calcium content (12–26 wt%) resulted in the fast crystallization of hydroxyapatite after immersion in SBF (<24 h). A thorough analysis allowed the uncoupling of the effect of composition and textural properties on bioactivity: the amount of calcium phosphate produced was mainly impacted by the composition, while HAp crystallization kinetics were strongly related to the specific surface area. Regarding copper-doped MBGNs, HAp crystallization was evidenced between 24 and 48 h due to a higher glass connectivity. Cu^2+^ was successfully released in SBF, and its concentration can easily be controlled by the copper content in the glass. Such structural analysis provides valuable insights for the design of tailored materials for bone tissue regeneration. Further studies will focus on biological effects (angiogenesis, bactericidity, and cytotoxicity) and on shaping capabilities of such spherical and non-agglomerated MBGNs.

## Figures and Tables

**Table 1:** Effective composition and textural properties of MBGNs and Cu-MBGN.

Sample	Effective Molar Ratio Ca/Si (EDS)	Effective Composition (wt%)	BET Specific Surface Area (m^2^/g)	Pore Volume (cm^3^/g)
MBGN-0.25	0.14 ± 0.01	88SiO_2_–12CaO *	909 ± 14	0.65
MBGN-0.5	0.22 ± 0.01	83SiO_2_–17CaO *	637 ± 9	0.50
MBGN-1	0.30 ± 0.01	78SiO_2_–22CaO *	431 ± 4	0.34
MBGN-2	0.37 ± 0.02	74SiO_2_–26CaO *	208 ± 1	0.22
Cu-MBGN	0.25 ± 0.01	75.6SiO_2_–15.5CaO–8.8CuO **	418 ± 4	0.24

Effective weight composition calculated either * from EDS measurements or ** from ICP-AES measurements.

**Table 2:** ^29^Si chemical shifts; relative populations (pop.) of Q^4^, Q^3^, and Q^2^ species; and coordination number (CN) of pristine and copper-doped MBGNs.

	Q^4^		Q^3^		Q^2^		
Sample	δ (ppm)	pop. (%)	δ (ppm)	pop. (%)	δ (ppm)	pop. (%)	CN
MBGN-0.5	–108.74	34	–98.00	50	–84.29	16	3.16
Cu-MBGN	–109.29	68	–98.39	32	–87.72	6	3.79
MBGN-1	–109.76	31	–98.59	50	–88.01	19	3.10

## Data Availability

The data presented in this study are available on request from the corresponding author.

## Supplementary Materials

The following supporting information can be downloaded at: https://www.mdpi.com/article/10.3390/ma16206690/s1. Figure S1: Size histogram of MBGN-1 (A) and Cu-MBGNs (B) fitted with log-normal functions; Figure S2: Textural properties (A,B) and XRD patterns (C) of Cu-MBGNs synthesized with a constant nominal molar Ca/Si ratio and various nominal molar Cu/Si ratios (△ CuO JCPDS 48-1548, ○ calcium silicate 29-0371, □ calcium carbonate 47-1743). The sample extensively characterized in the present article is evidenced by an arrow; Figure S3: TEM images of MBGNs synthesized with [CTAB] = 1 mM and an initial molar Ca/Si ratio = 0.5; Figure S4: XRD patterns and FTIR spectra of Cu-MBGNs with various initial molar Cu/Si ratios, after 24 h of immersion in SBF (◊ hydroxyapatite JCPDS 09-0432). The sample described in the present paper is evidenced by an arrow; Figure S5: Released copper after 24 h of immersion in SBF for various Cu-doped MBGNs samples; Figure S6: EDS spectra (A,D) of Cu-MBGNs identified on STEM images before (B) and after (C) 4 days in SBF.
