# Supplementary material for: Correlating the Effect of Composition and Textural Properties on Bioactivity for Pristine and Copper-Doped Binary Mesoporous Bioactive Glass Nanoparticles
Source: Materials (Basel). 2023 Oct 14;16(20):6690. doi: 10.3390/ma16206690 (PMC10608725; doi:10.3390/ma16206690)
Supplement: Supplementary file 1 [file materials-16-06690-s001.zip › materials-2653141-supplementary.pdf]

Electronic Supporting Information (ESI)

Correlating the impact of composition and textural properties on bioactivity for pristine and copper doped binary mesoporous bioactive glass nanoparticles.

F. Vergnaud,[a] B. Mekonnen,[a] A. El Abbassi,[a] C. Vichery,[a] J.-M. Nedelec[a]

[a] Université Clermont Auvergne, Clermont Auvergne INP, CNRS, ICCF, F-63000 Clermont-Ferrand, France. E-mail: charlotte.vichery@sigma-clermont.fr

Size histograms of MBGNs and Cu-MBGNs

TEM images were analyzed with the ImageJ software to determine particles size distribution. The histograms (Fig. S1) were fitted with a log-normal function defined as:

$$P(d)=\frac{1}{\sqrt{2\pi}\cdot d\cdot\sigma_d}\cdot\exp\left(-\frac{(\ln(d)-\mu)^2}{2\sigma_d^2}\right)$$

Mean diameter d_m and size distribution σ were extracted thanks to the following expressions:

$$d_m=\exp\left(\mu+\frac{\sigma_d^2}{2}\right) \quad \text{and} \quad \sigma=\sqrt{(\exp(\sigma_d^2)-1)\cdot d_m^2}$$

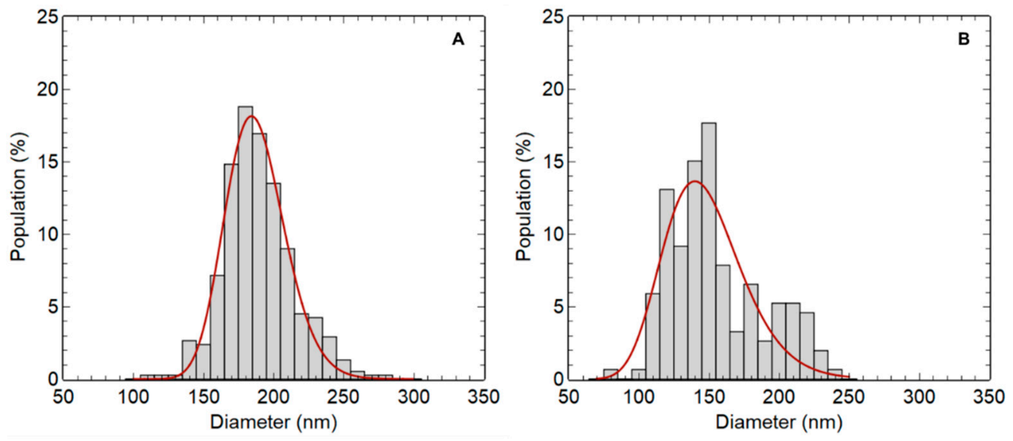

Figure S1. Size histogram of MBGN-1 (A) and Cu-MBGNs (B) fitted with log-normal functions.

Cu-MBGNs synthesis with various amounts of copper

For a constant initial molar ratio Ca/Si = 1, Cu-doped MBGNs were synthesized with various amounts of copper by controlling the copper nitrate amount added in the reaction medium (Cu/Si ratio ranging from 0.002 to 0.2).

The textural properties deduced from N_2 sorptometry do not seem to be strongly affected by the Cu/Si ratio variation (Fig. S2A and S2B). On the other hand, XRD patterns (Fig. S2C) show the crystallization of copper oxide (CuO) above a certain amount of introduced copper nitrate. The diffraction peaks are more intense as the Cu/Si ratio increases. This could be explained by the electrostatic repulsion between Ca^{2+} and Cu^{2+} ions which would induce the formation and separation of crystalline phases, hence the progressive appearance of calcium silicate in parallel.

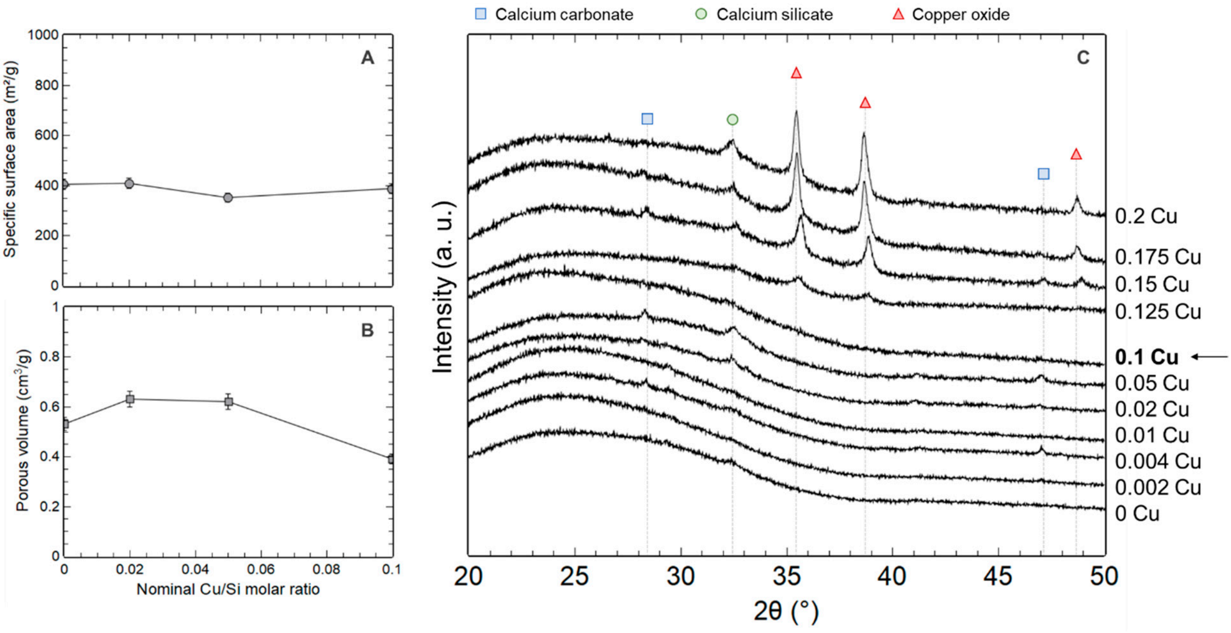

Figure S2. Textural properties (A, B) and XRD patterns (C) of Cu-MBGNs synthesized with a constant nominal molar Ca/Si ratio and various nominal molar Cu/Si ratios (△ CuO JCPDS 48-1548, ○ calcium silicate 29-0371, □ calcium carbonate 47-1743). The sample extensively characterized in the present article is evidenced by an arrow.

MBGNs synthesis with [CTAB] = 1 mM and Ca/Si = 0.5

For an initial Ca/Si ratio of 0.5, when the CTAB concentration is low (1 mM, *i.e.,* 109 mg), the NPs morphology is strongly affected, even with a late addition of the calcium precursor (3 h after TEOS). The spherical particles seem to break up into smaller particles, probably due to a collapse of the structure following the interactions between calcium ions and surfactant. Note that the specific surface area is strongly reduced (212 m^2/g here, compared to 637 m^2/g for the MBGN-0.5 sample), probably due to a strong agglomeration between these smaller particles.

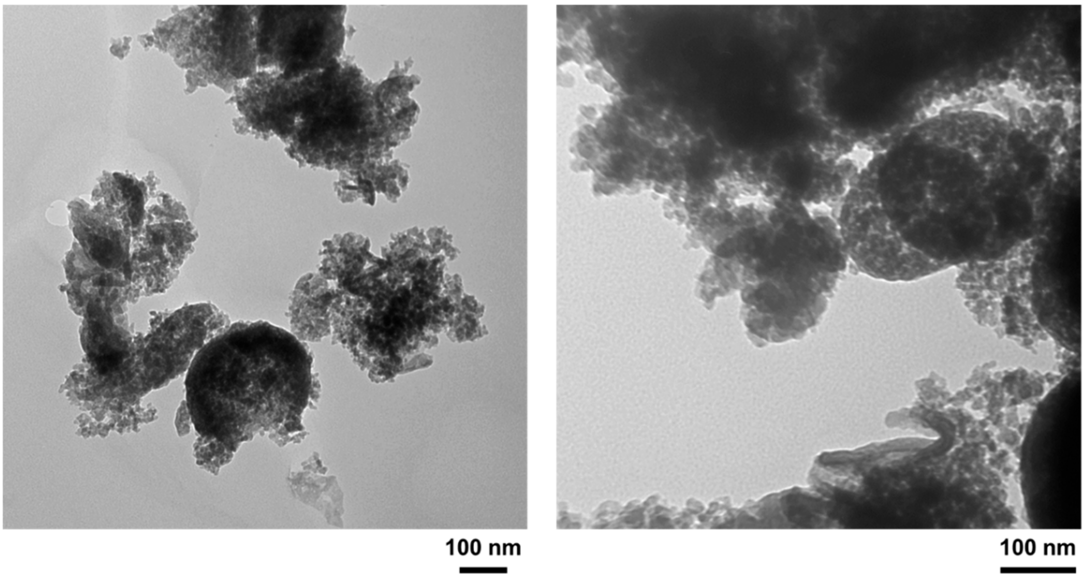

Figure S3. TEM images of MBGNs synthesized with [CTAB] = 1 mM and an initial molar Ca/Si ratio = 0.5

Cu-MBGNs reactivity for low copper content

HAp crystallization is evidenced as soon as 24 h of immersion in SBF for lower copper contents (0 to 0.01 Cu), while the sample described in the present study (0.1 Cu) does require 24 to 48 h to show HAp crystallization. As the specific surface area is not significantly affected by copper content (Fig. S2A), the HAp formation inhibition seems to be solely due to the composition.

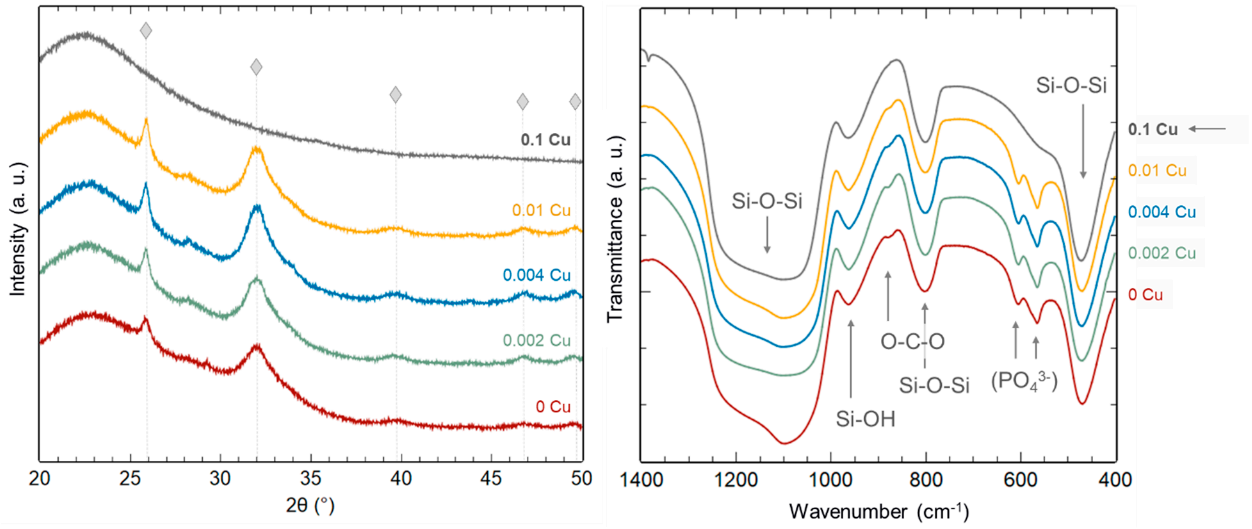

Figure S4. XRD patterns and FTIR spectra of Cu-MBGNs with various initial molar Cu/Si ratios, after 24 h of immersion in SBF (◊ hydroxyapatite JCPDS 09-0432). The sample described in the present paper is evidenced by an arrow.

Copper release from various Cu-doped MBGNs

Samples with various copper amounts were assessed in SBF following the same protocol as the one for the sample described in the main study (1 mg of powder for 1 mL of SBF). The copper quantity released in SBF is proportional to the nominal Cu/Si ratio, indicating that desired biological effect can be finely tuned by controlling Cu-MBGNs composition.

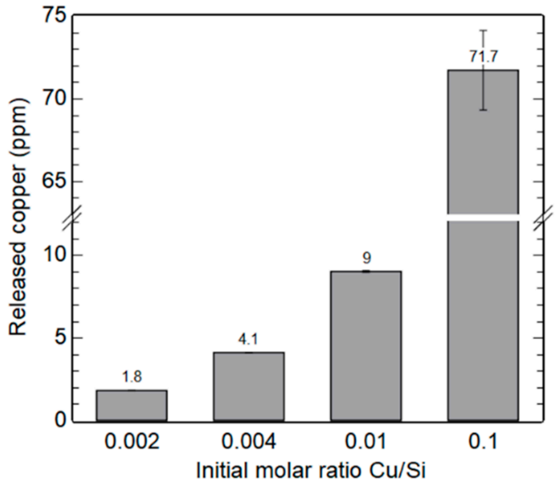

Figure S5. Released copper after 24 h of immersion in SBF for various Cu-doped MBGNs samples.

Elemental analysis of Cu-MBGNs single particles

EDS spectroscopy was performed on Cu-MBGNs using a STEM. Spectra were acquired by focusing on single spherical MBGNs, before and after immersion in SBF.

The measured Ca/Si ratio on Cu-MBGNs before SBF is underestimated compared to the value measured by EDS on pellets with a SEM, or by ICP-AES (0.13 ± 0.05 versus 0.25 ± 0.01). This may be due to the finite volume involved in such EDS measurements on single nanoparticles deposited on a membrane and/or to the lower precision of EDS in this configuration. On the other hand, the Cu/Si ratio is overestimated (0.69 ± 0.09 versus 0.09 ± 0.01).

Using an identical measurement method with STEM, a decrease in Ca and Cu levels is evidenced after 4 days of immersion: no calcium is detected (Fig. S6D) and a measured Cu/Si ratio of 0.26 could indicate a loss up to 63 % of the initial amount of copper due to ionic release.

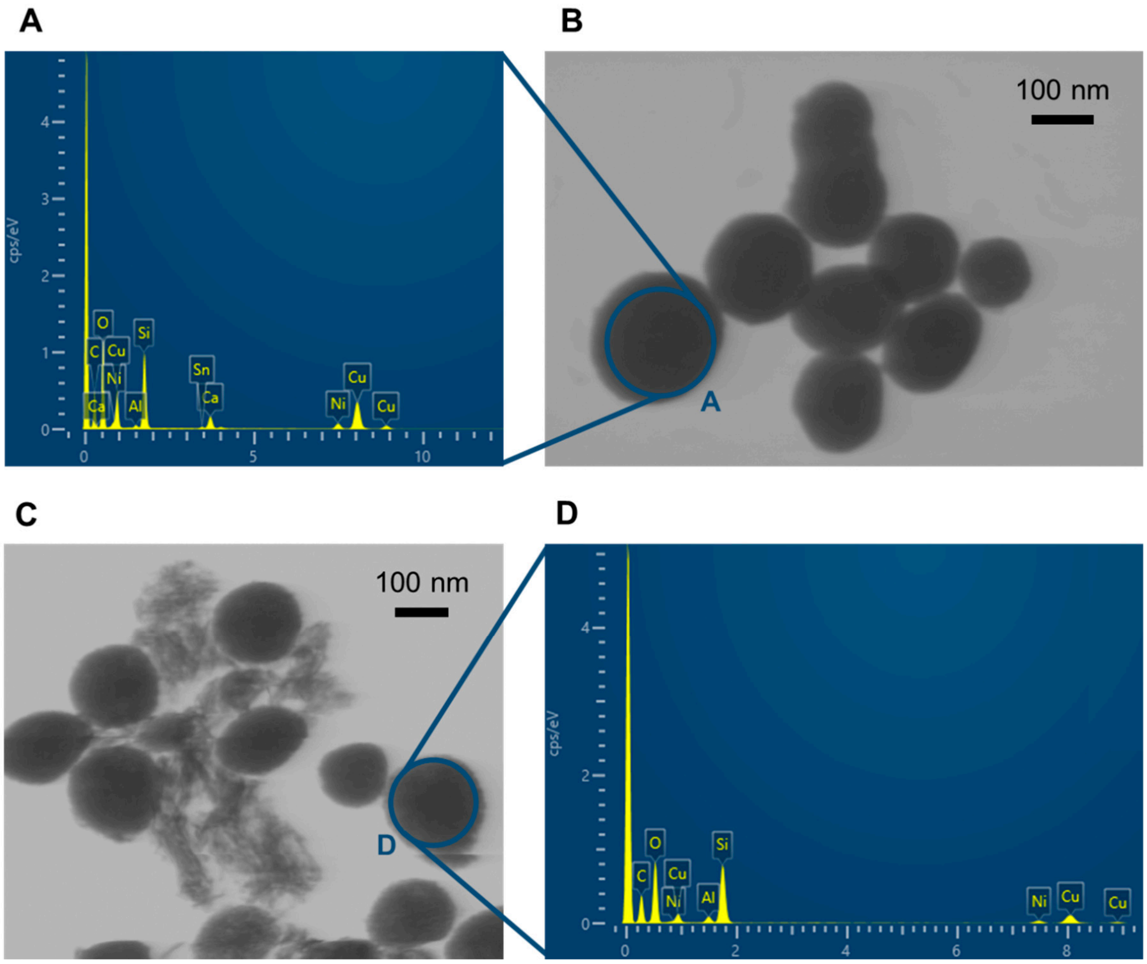

Figure S6. EDS spectra (A, D) of Cu-MBGNs identified on STEM images before (B) and after (C) 4 days in SBF
